# Infections with the SARS-CoV-2 Omicron variant show a similar outcome as infections with the previous variants in patients with hematologic malignancies

**DOI:** 10.1007/s00277-022-04833-8

**Published:** 2022-04-07

**Authors:** Ryutaro Taenaka, Teppei Obara, Kentaro Kohno, Kenichi Aoki, Ryosuke Ogawa

**Affiliations:** Department of Hematology and Oncology, JCHO Kyushu Hospital, 1-8-1 Kishinoura, Yahatanishi-ku, Kitakyushu, Fukuoka Japan

Dear Editor,

By October 2021, high mortality rates had been reported in patients with hematologic malignancies (HM) who contracted COVID-19 [1–3]. The B.1.1.529 variant, named Omicron, emerged in November 2021 and replaced Delta as the dominant variant worldwide. The Omicron variant has been reported to cause lower rates of hospitalization, severe disease, and mortality compared with other variants [4, 5], but the prognosis in patients with HM is unclear. Our study reports our experience of a nosocomial outbreak in nine patients with HM who were infected with the Omicron variant at our hospital.

An outbreak of COVID-19 occurred in our hematology ward in February 2022 following the admission of a pre-symptomatic patient. A total of nine patients were diagnosed with COVID-19 by SARS-CoV-2 reverse quantitative polymerase chain reaction testing of nasopharyngeal swabs. The nasopharyngeal swabs of eight of the nine patients were tested for S-gene targeting failure (SGTF); these eight patients had SGTF. All nine cases were diagnosed as Omicron variant infection because the prevalence of Omicron was greater than 95% in Kitakyushu City at that time, and all infections had occurred within a few days of each other.

The mean (± standard deviation) age of the nine patients was 74 ± 7 years (range, 61 to 85 years), and six (66.7%) were male. All subjects had active HM: 3 (33.3%) with myeloproliferative disease, 4 (44.4%) with malignant lymphoma, and 2 (22.2%) with multiple myeloma. Six (66.7%) patients had received two doses of vaccine more than 6 months ago. All survivors were followed up for at least 17 days. According to the National Institutes of Health severity classification [6], one (11.1%) patient was asymptomatic, and illness was mild in three (33.3%), moderate in two (22.2%), and critical in three (33.3%). Each attending physician decided the treatment options among sotrovimab, remdesivir, dexamethasone, high-dose corticosteroids, baricitinib, and tocilizumab, taking into account the severity of COVID-19, organ damage, and comorbidities. Two (22.2%) deaths occurred, and these were attributed to COVID-19. Two of the survivors remain critically ill and their situation is life-threatening (Table 1).

**Table 1:** Patient characteristics

Age/sex	Vaccine	Disease	Status	HM therapy	Immunosuppressant	COVID-19 severity	COVID-19 therapy	Outcome	Cause of death
61/M	2 doses	AITL	ND	A-CHP	None	Mild	Sotrovimab	Alive	
65/M	0 dose	AML	R/R	HaploPBSCT	Prednisolone, tacrolimus	Critical	Remdesivir, HD-CCSs	Alive	
74/M	2 doses	DLBCL	ND	R-THP-COP	None	Asymptomatic	None	Alive	
74/F	0 dose	AML	R/R	5-Aza + Ven	None	Mild	Sotrovimab	Alive	
76/M	0 dose	MM	R/R	PACE	None	Critical	Sotrovimab	Alive	
76/F	2 doses	MM	R/R	Kd	Hydrocortisone	Moderate	Sotrovimab, DEX	Dead on day 8	Aspiration of sputum
78/F	2 doses	ALK-ALCL	R/R	Tucidinostat	None	Moderate	None	Alive	
81/M	2 doses	SS	R/R	DeVIC	Hydrocortisone	Critical	DEX	Dead on day 12	Respiratory failure
85/M	2 doses	MDS	R/R	5-Aza	Prednisolone	Mild	Sotrovimab	Alive	

*HM*, hematologic malignancies; *M*, male; *F*, female; *AITL*, angioimmunoblastic T-cell lymphoma; *AML*, acute myeloid leukemia; *DLBCL*, diffuse large B-cell lymphoma; *MM*, multiple myeloma; *ALK-ALCL*, anaplastic lymphoma kinase–negative anaplastic large cell lymphoma; *SS*, Sezary syndrome; *MDS*, myelodysplastic syndrome; *ND*, newly diagnosed; *R/R*, relapsed and refractory; *A-CHP*, brentuximab vedotin, cyclophosphamide, doxorubicin, and prednisolone; *HaploPBSCT*, haploidentical peripheral blood stem cell transplantation; *R-THP-COP*, rituximab, pirarubicin, cyclophosphamide, vincristine, and prednisolone; *5-Aza*, azacitidine; *Ven*, venetoclax; *PACE*, cisplatin, doxorubicin, cyclophosphamide, and etoposide, *Kd*, carfilzomib and dexamethasone, *DeVIC*, dexamethasone, etoposide, ifosfamide, and carboplatin; *HD-CCSs*, high-dose corticosteroids; *DEX*, dexamethasone

A study that analyzed data from patients with HM who were infected with COVID-19 in the year after March 2020 (before the appearance of the Omicron variant), 63.8% of cases had a severe or critical illness and 22.2% died due to COVID-19 [3]. Compared with that study, the present report showed a similar mortality rate despite the lower severity of the disease. Furthermore, both of the critical patients were in a life-threatening situation at the time that this report was submitted, meaning that the mortality rate could possibly increase further. Side effects of treatment for HM and comorbidities may increase mortality in hospitalized patients with HM. In fact, a patient with dysphagia died from aspiration of sputum despite having only moderate severity of the disease.

In conclusion, although the Omicron variant may be less severe than previous variants, the mortality rate of hospitalized HM patients infected with the Omicron variant remains high. It is important that infection control measures are not neglected despite the development of vaccines and the implementation of treatment measures.
